# Supplementary material for: CHD Risk Minimization through Lifestyle Control: Machine Learning Gateway
Source: Sci Rep. 2020 Mar 5;10:4090. doi: 10.1038/s41598-020-60786-w (PMC7058059; doi:10.1038/s41598-020-60786-w)
Supplement: Supplementary file 1 — Supplementary information. [file 41598_2020_60786_MOESM1_ESM.docx]

**CHD Risk Minimization through Lifestyle Control: Machine Learning Gateway**

**(ONLINE ONLY SUPPLEMENTARY MATERIAL: Real Data vs Machine Learned data – ANOVA)**

**Xi He^1^, B. Rajeswari Matam^1^, Srikanth Bellary^2,3^ , Goutam Ghosh^4^ and Amit K Chattopadhyay^1^**

^1^Aston University, Systems Analytics Research Institute, Mathematics, Birmingham B4 7ET, UK

^2^University Hospitals Birmingham NHS Foundation Trust, UK

^3^Aston University, School of Life and Health Sciences, Aston Triangle, Birmingham B4 7ET, UK

^4^Vice Chancellor, GIET University, Gunupur 765022, Dt. Rayagada (Odisha), India

Corresponding Author: Amit K Chattopadhyay

Email: a.k.chattopadhyay@aston.ac.uk

Table summarising the combined male-vs-female statistics to better understand the representation and possible skew, if any, that is present in the data.

| **United Kingdom**  **SDR, Coronary Heart Disease (CHD) Death Rate by 100,000 inhabitants** | | | | | | |
| --- | --- | --- | --- | --- | --- | --- |
|  | *All causes death rate* | | *CHD death rate* | | *CHD/All causes death rate* | |
| *Year* | *Male* | *Female* | *Male* | *Female* | *Male* | *Female* |
| 1990 | 1055.87 | 659.77 | 309.09 | 145.41 | 0.2927 | 0.2204 |
| 1991 | 1042.74 | 656.32 | 304.95 | 145.77 | 0.2925 | 0.2221 |
| 1992 | 1009.20 | 637.20 | 292.93 | 140.12 | 0.2903 | 0.2199 |
| 1993 | 1028.00 | 654.59 | 290.70 | 138.78 | 0.2828 | 0.2120 |
| 1994 | 972.35 | 621.54 | 266.46 | 127.29 | 0.2740 | 0.2048 |
| 1995 | 979.75 | 629.82 | 259.90 | 123.07 | 0.2653 | 0.1954 |
| 1996 | 952.13 | 616.57 | 247.89 | 117.22 | 0.2604 | 0.1901 |
| 1997 | 925.10 | 608.79 | 232.72 | 110.77 | 0.2516 | 0.1820 |
| 1998 | 912.56 | 603.04 | 225.84 | 108.31 | 0.2475 | 0.11796 |
| 1999 | 902.49 | 602.80 | 214.66 | 101.74 | 0.2379 | 0.1688 |
| 2000 | 858.60 | 574.90 | 199.92 | 94.38 | 0.2328 | 0.1642 |
| 2001 | 838.63 | 565.76 | 191.15 | 90.56 | 0.2279 | 0.1601 |
| 2002 | 828.18 | 562.92 | 182.31 | 87.04 | 0.2201 | 0.1546 |
| 2003 | 817.66 | 567.18 | 173.97 | 83.46 | 0.2128 | 0.1471 |
| 2004 | 774.41 | 536.81 | 160.62 | 75.99 | 0.2074 | 0.1416 |
| 2005 | 752.40 | 527.44 | 150.44 | 70.79 | 0.1999 | 0.1342 |
| 2006 | 727.20 | 508.57 | 138.20 | 65.10 | 0.1900 | 0.1280 |
| 2007 | 711.61 | 501.79 | 131.77 | 61.05 | 0.1852 | 0.1217 |
| 2008 | 700.40 | 499.39 | 124.07 | 58.01 | 0.1771 | 0.1162 |
| 2009 | 670.69 | 472.64 | 115.60 | 52.22 | 0.1724 | 0.1105 |
| 2010 | 654.69 | 467.36 | 111.12 | 49.45 | 0.1697 | 0.1058 |
| 2011 | 630.56 | 451.55 | 100.88 | 44.37 | 0.1600 | 0.0983 |
| 2012 | 631.45 | 460.36 | 97.91 | 44.05 | 0.1551 | 0.0957 |
| 2013 | 630.83 | 457.16 | 96.48 | 42.53 | 0.1529 | 0.0930 |

**Table 1: Raw real datasets of UK CHD death rate**

| **United Kingdom – Databases for 6 parameters** | | | | | | | | |
| --- | --- | --- | --- | --- | --- | --- | --- | --- |
|  | *Alcohol*  *consumed* | *Cheese*  *consumed* | *Smoking habit* | | *Mean Systolic*  *Blood Pressure* | | *Cereal*  *consumed* | *Fruit-Veg*  *consumed* |
| *Year* |  |  | *Male* | *Female* | *Male* | *Female* |  |  |
| 1990 | 9.96 | 7.74 | 31.00 | 29.00 | 133.20 | 127.30 | 92.97 | 164.43 |
| 1991 | 10.01 | 8.31 | 30.90 | 29.06 | 133.40 | 127.50 | 93.02 | 163.83 |
| 1992 | 9.65 | 8.46 | 29.00 | 28.00 | 133.50 | 127.60 | 95.25 | 168.64 |
| 1993 | 9.45 | 8.29 | 29.98 | 28.07 | 133.60 | 127.60 | 92.88 | 168.47 |
| 1994 | 9.41 | 8.34 | 28.00 | 26.00 | 133.60 | 127.50 | 92.61 | 162.46 |
| 1995 | 9.70 | 8.32 | 29.00 | 26.00 | 133.50 | 127.40 | 93.83 | 155.80 |
| 1996 | 9.75 | 9.78 | 29.00 | 28.00 | 133.40 | 127.20 | 96.18 | 162.69 |
| 1997 | 9.97 | 10.08 | 8.16 | 26.09 | 133.20 | 126.90 | 101.09 | 164.03 |
| 1998 | 10.14 | 9.54 | 28.00 | 26.00 | 133.00 | 126.50 | 107.39 | 178.02 |
| 1999 | 10.16 | 9.83 | 27.25 | 25.10 | 132.70 | 126.10 | 107.56 | 178.71 |
| 2000 | 10.59 | 9.21 | 29.00 | 25.00 | 132.40 | 125.70 | 107.80 | 171.82 |
| 2001 | 10.91 | 10.05 | 28.00 | 26.00 | 132.10 | 125.20 | 104.57 | 182.79 |
| 2002 | 11.44 | 9.46 | 27.00 | 25.00 | 131.70 | 124.60 | 114.47 | 183.63 |
| 2003 | 11.85 | 9.63 | 28.00 | 24.00 | 131.30 | 124.10 | 114.39 | 207.90 |
| 2004 | 12.22 | 9.96 | 26.00 | 23.00 | 130.90 | 123.50 | 111.92 | 206.49 |
| 2005 | 12.05 | 10.58 | 25.00 | 23.00 | 130.40 | 122.90 | 113.30 | 222.90 |
| 2006 | 11.61 | 10.84 | 23.00 | 21.00 | 130.00 | 122.30 | 112.34 | 232.44 |
| 2007 | 11.84 | 11.3 | 22.00 | 20.00 | 129.60 | 121.80 | 113.59 | 218.51 |
| 2008 | 11.47 | 10.93 | 22.00 | 21.00 | 129.20 | 121.20 | 115.79 | 227.20 |
| 2009 | 10.79 | 10.79 | 22.00 | 20.00 | 128.70 | 120.60 | 114.53 | 213.78 |
| 2010 | 10.88 | 11.09 | 21.00 | 20.00 | 128.20 | 120.10 | 114.95 | 216.12 |
| 2011 | 10.68 | 10.73 | 21.00 | 19.00 | 127.80 | 119.50 | 114.21 | 222.64 |
| 2012 | 10.42 | 11.17 | 22.00 | 19.00 | 127.30 | 118.90 | 1114.89 | 218.91 |
| 2013 | 10.32 | 11.33 | 22.00 | 17.00 | 126.90 | 118.40 | 115.85 | 224.40 |

**Table 2: UK raw real datasets of 6 life-style parameters**

**ANOVA Statistics of Lifestyle parameters:**

Analysis of Variance (ANOVA) based statistical models and their associated estimation procedures have been used to analyse the differences across the entire lifestyle parametric regime of our ‘synthetic data’.

| *Regression Statistics df SS MS F Significance F* |
| --- |
| Multiple R 0.603 Regression 1 6.298 6.298 12.549 0.002  R Square 0.363 Residual 22 11.041 0.502  Adjusted R Square 0.334 Total 23 17.339  Standard Error 0.708  Observations 24 |

**Table 3: Regression and ANOVA test Statistics of UK alcohol consumption**

| *Regression Statistics df SS MS F Significance F* |
| --- |
| Multiple R 0.932 Regression 1 24.140 24.140 145.380 3.627E-11  R Square 0.869 Residual 22 3.653 0.166  Adjusted R Square 0.863 Total 23 27.793  Standard Error 0.407  Observations 24 |

**Table 4: Regression and ANOVA test Statistics of UK cheese consumption**

| *Regression Statistics df F Significance F* |
| --- |
| *Males Females* Regression-Male 1 166.216 9.905E-12  Multiple R 0.940 0.970 Residual-Male 22  R Square 0.883 0.941 Total-male 23  Adjusted R Square 0.878 0.938 Regression-Female. 1 351.704 5.090E-15  Standard Error 1.183 0.880 Residual-Female 22  Observations 24 24 Total-Female 23 |

**Table 5: Regression and ANOVA test Statistics of UK regular daily smokers**

| *Regression Statistics df F Significance F* |
| --- |
| *Males Females* Regression-Male 1 256.2233 1.3195E-13  Multiple R 0.960 0.973 Residual-Male 22  R Square 0.921 0.946 Total-male 23  Adjusted R Square 0.917 0.944 Regression-Female. 1 388.773 1.794E-15  Standard Error 0.648 0.8744 Residual-Female 22  Observations 24 24 Total-Female 23 |

**Table 6: Regression and ANOVA test Statistics of UK mean systolic blood pressure**

| *Regression Statistics df SS MS F Significance F* |
| --- |
| Multiple R 0.921 Regression 1 1609 1609 122.392 1.863E-10  R Square 0.848 Residual 22 289 13  Adjusted R Square 0.841 Total 23 1899  Standard Error 3.626  Observations 24 |

**Table 7: Regression and ANOVA test Statistics of UK cereals supply quantities**

| *Regression Statistics df SS MS F Significance F* |
| --- |
| Multiple R 0.912 Regression 1 13437 13437 108.069 5.932E-10  R Square 0.831 Residual 22 2735 124  Adjusted R Square 0.823 Total 23 16172  Standard Error 11.151  Observations 24 |

**Table 8: Regression and ANOVA test Statistics of UK fruits and vegetables supply quantities**

**DATA VISUALIZATION RESULTS:**

Figure 3 shows the PCA visualization of (a) real, (b) synthetic and (c) real-synthetic datasets obtained for all 6 variables (i.e. alcohol, cheese, smoking, SBP, cereals3, fruits and vegs) for males (left) and females (right) respectively.


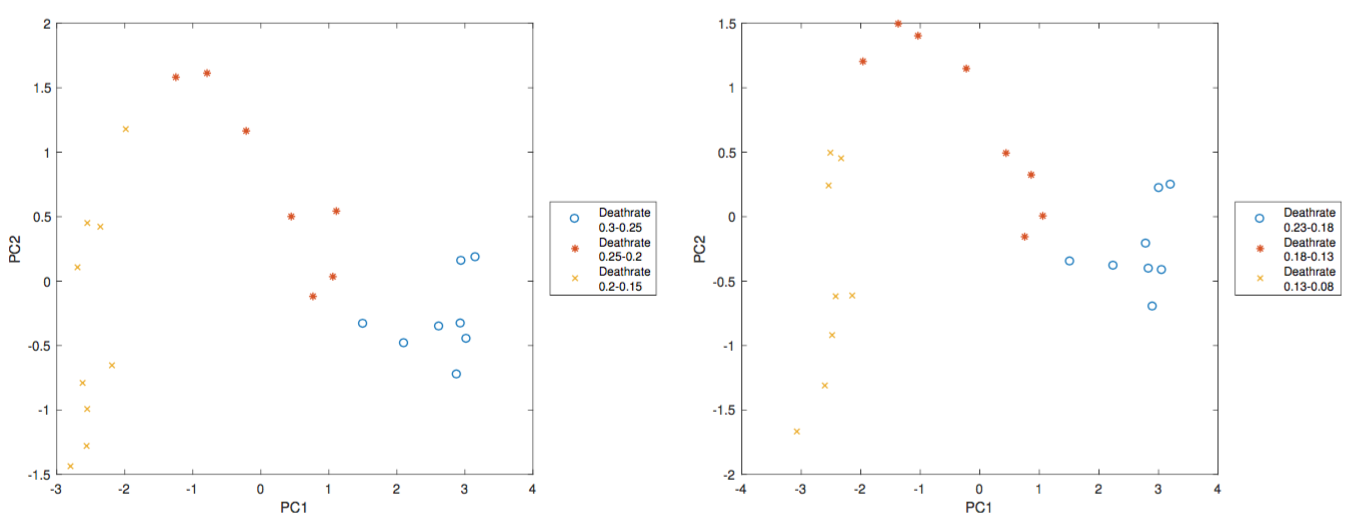


**Figure 3(a): PCA 6-dimensional visualisation of real datasets**


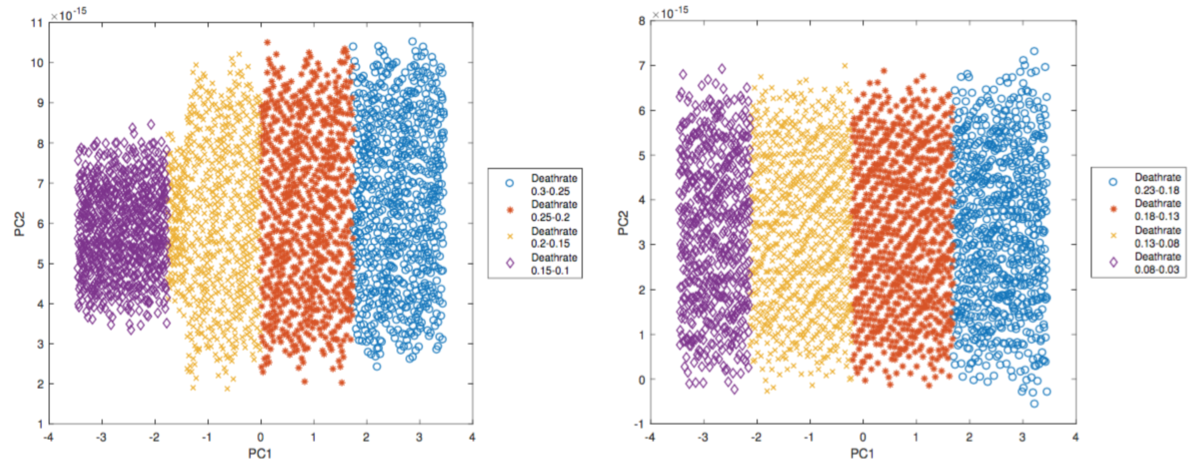


**Figure 3(b): PCA 6-dimensional visualisation of synthetic datasets**


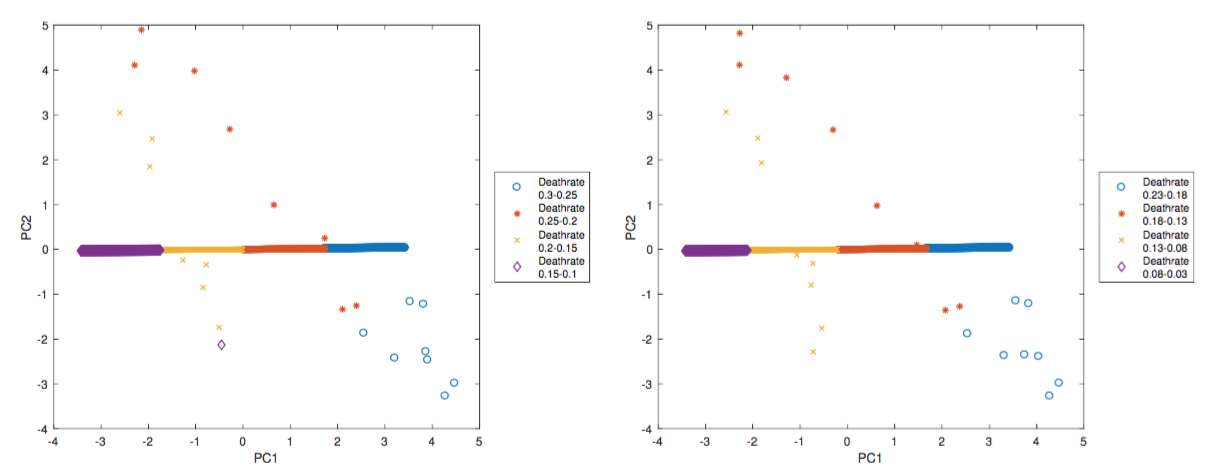


**Figure 3(c): PCA 6-dimensional visualisation of real-synthetic datasets**

Figure 4, on the other hand, shows the nonlinear NSC visualization of (a) real, (b) synthetic and (c) real-synthetic datasets obtained for all the 6 variables (i.e. alcohol, cheese, smoking, SBP, cereals3, fruits and vegs) for males (left) and females (right) respectively.


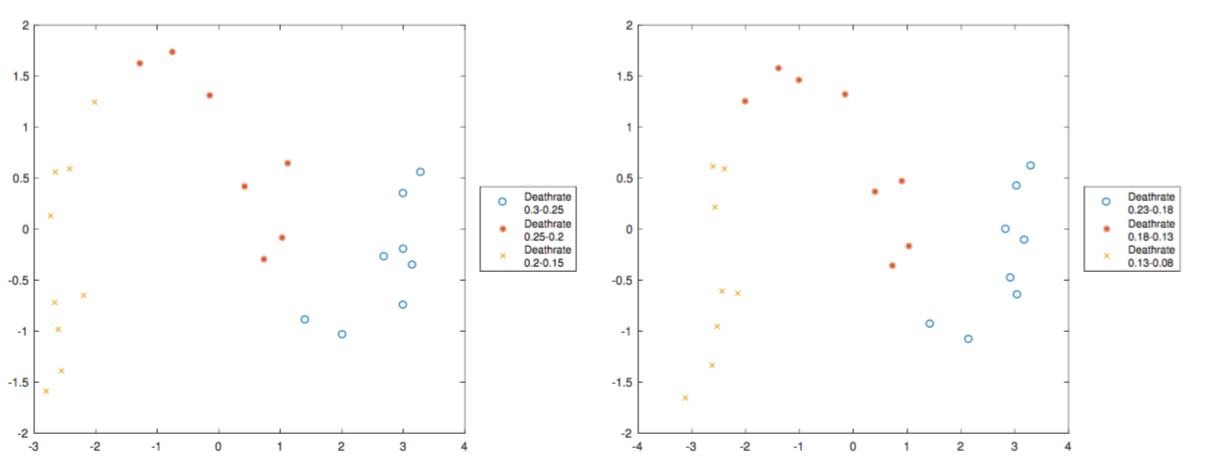


**Figure 4(a): NSC 6-dimensional visualisation of real datasets**


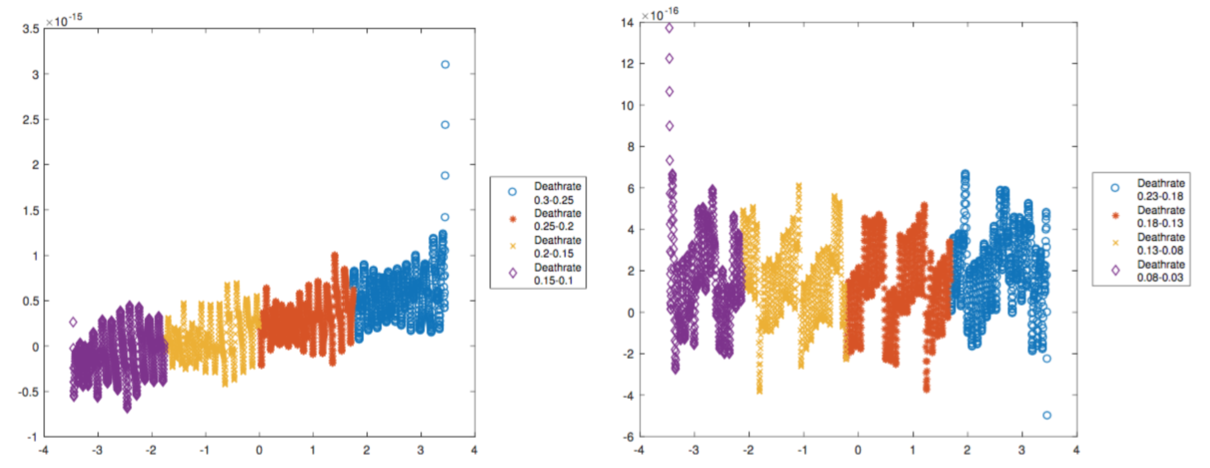


**Figure 4(b): NSC 6-dimensional visualisation of synthetic datasets**


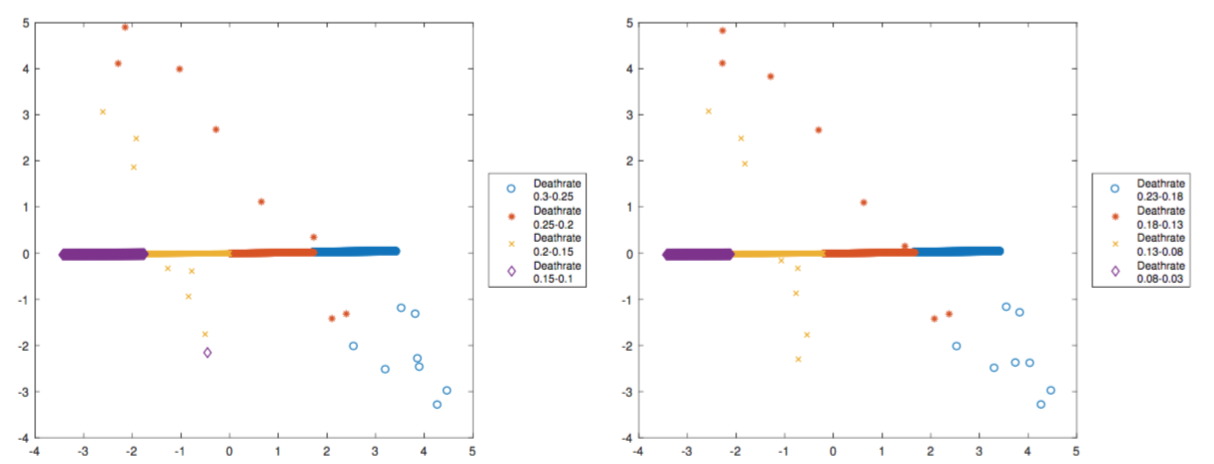


**Figure 4(c): NSC 6-dimensional visualisation of real-synthetic datasets**

Table 6 lists the variance of the six variables in decreasing order, the element with the highest variance contributes the highest “uncertainty” to the prediction.

|  | **Alcohol** | **Cheese** | **Smoking** | | **SBP** | | **Cereals** | **Fruits & Vegs** |
| --- | --- | --- | --- | --- | --- | --- | --- | --- |
|  |  |  | **Males** | **Females** | **Males** | **Females** |  |  |
| Variance | 0·7539 | 1·2084 | 11·4471 | 12·5928 | 5·0867 | 9·8910 | 82·5475 | 703·1390 |

**Table 9: Variance of 6 variables**
